# Virus Infections and Host Metabolism—Can We Manage the Interactions?

**DOI:** 10.3389/fimmu.2020.594963

**Published:** 2021-02-03

**Authors:** Deepak Sumbria, Engin Berber, Manikannan Mathayan, Barry T. Rouse

**Affiliations:** ^1^ Department of Biomedical and Diagnostic Sciences, College of Veterinary Medicine, The University of Tennessee, Knoxville, TN, United States; ^2^ Department of Virology, Faculty of Veterinary Medicine, Erciyes University, Kayseri, Turkey; ^3^ Center for Drug Discovery and Development, Sathyabama Institute of Science and Technology, Chennai, India

**Keywords:** virus, metabolism, interferon, diabetes, obesity, short chain fatty acids, metabolic blockers

## Abstract

When viruses infect cells, they almost invariably cause metabolic changes in the infected cell as well as in several host cell types that react to the infection. Such metabolic changes provide potential targets for therapeutic approaches that could reduce the impact of infection. Several examples are discussed in this review, which include effects on energy metabolism, glutaminolysis and fatty acid metabolism. The response of the immune system also involves metabolic changes and manipulating these may change the outcome of infection. This could include changing the status of herpesviruses infections from productive to latency. The consequences of viral infections which include coronavirus disease 2019 (COVID-19), may also differ in patients with metabolic problems, such as diabetes mellitus (DM), obesity, and endocrine diseases. Nutrition status may also affect the pattern of events following viral infection and examples that impact on the pattern of human and experimental animal viral diseases and the mechanisms involved are discussed. Finally, we discuss the so far few published reports that have manipulated metabolic events *in-vivo* to change the outcome of virus infection. The topic is expected to expand in relevance as an approach used alone or in combination with other therapies to shape the nature of virus induced diseases.

## Introduction

Viruses are obligate intracellular parasites and usually cause changes and often times death to cells that support their replication. In the infected host, this may manifest as disease which in some cases is the direct result of virus replication events in target cells. In other circumstances, clinical consequences of viral infection are attributed, fully or in part, to the host immune response to infected cells, or to extracellular virus (1–3). Whatever the means by which viruses act as pathogens, the infection results in changes in metabolism in the cells they infect and also in multiple cell types of the host that react to the infection. In addition, innate responses set off by viral infections, such as interferons type I and II, also act to modulate some aspects of metabolism (4). The consequences of many viral infections can differ in hosts that have abnormal metabolism such as diabetes mellitus (DM). This has become an important issue in the case of coronavirus disease 2019 (COVID-19) infection where diabetics are more likely to suffer from more severe disease (5). In addition, accumulating evidence shows that the state of nutrition can influence the outcome of a viral infection by causing changes in one or more aspect of metabolism (6). All of these observations imply that adjusting metabolic events during the course of a viral infection could represent a valuable approach to reshape the outcome of infections (7, 8). For example, when disease lesions result from immune-inflammatory reactions to infected tissues, changing the metabolic environment that limits the function of inflammatory cell subsets may change the reaction from being highly tissue damaging to one that acts to resolve lesions (8). In this brief review, we discuss evidence showing that manipulating metabolism can represent a useful approach to control the outcome of a virus infection.

## Virus Infection Usually Imposes Metabolic Changes in Target Cells

Viruses themselves are metabolically inert and must rely on metabolic events in the cell to generate its component parts and to replicate new viral copies. Oftentimes, the cell at the time of infection is in a quiescent state, but the infection acts to change the cell’s metabolic activity. There are multiple mechanisms by which a virus infection can induce metabolic changes in infected cell and these are listed in **Table 1**. A common consequence of infection by many viruses is to induce high glucose metabolism (causing aerobic glycolysis, the so-called Warburg effect) in cells and to change the nature of lipid metabolism usually from fatty acid oxidation (FAO) to fatty acid synthesis (FAS) (16, 22). Increased FAS is particularly necessary for those viruses that are enveloped. With some viruses, infection only occurs in cells that are already metabolically active with the infection often serving to downregulate one or more metabolic events. This state of affairs occurs with HIV, which preferentially infects cells that are in an activated metabolic state (23). Accordingly, HIV infects T cells undergoing the highest levels of metabolic activity, which includes elevated aerobic glycolysis (24), TCA (tricarboxylic acid cycle) cycle activity, oxidative phosphorylation (OXPHOS), and glutaminolysis (25–28). The result is cell destruction and the onset of immunosuppression. The multiple metabolic events that can be set into play by different types of virus infection are discussed in detail in an excellent recent review by Eisenreich et al. (29).

**Table 1:** Metabolic alteration in various virus infection.

Virus	Nucleic acid	Cells used	Metabolism targeted	Action	Reference
Adeno virus	DNA	Non-tumorigenic epithelial cell line (MCF10A cells)	Glycolysis	upregulated	(9)
HCMV		MRC-5 (human fetal lung) fibroblasts			(10)
EBV		Nasopharyngeal carcinoma cells			(11)
KSHV		Human B-cell non-Hodgkin lymphomas and primary effusion lymphoma			(12)
HCV	RNA	U-2 OS human osteosarcoma-derived tetracycline-regulated cell line			(13)
HIV		Primary CD4+ T cells from donor			(14)
DENV		Primary dermal fibroblast normal; human, neonatal cells			(15)
IAV		Madin-Darby canine kidney cells			(16)
HCMV	DNA	Human foreskin fibroblasts	Glutaminolysis		(17)
KSHV		Tert-immortalized microvascular endothelial cells			(18)
Vaccinia virus		Primary human foreskin fibroblasts			(19)
HCMV	DNA	MRC-5 fibroblasts	Fatty acid synthesis		(10)
KSHV		Human B-cell non-Hodgkin lymphomas and primary effusion lymphoma			(12)
HCV	RNA	Huh-7 cell			(20, 21)
DENV		Human embryonic lung cells			(22)

EBV, Epstein-Barr virus; IAV, influenza A virus; KSHV, Kaposi’s sarcoma-associated herpesvirus; DENV, dengue virus; HCV, hepatitis C virus; HCMV, human cytomegalovirus.

Curiously, a wide range of mechanisms are known whereby viruses influence metabolic events in their target cells. Of particular interest is the variable events that occur following infection with different flaviviruses. With dengue virus infection, for example, the virus taps into the host cell’s lipid reserves which are held in lipid droplets. The droplets are broken down by an autophagy type mechanism releasing fatty acids. These undergo oxidation which fuels the TCA cycle providing ATP and TCA intermediates needed for viral replication (30).

The Dengue related virus Zika (ZIKV) employs an interesting metabolic manipulation, although only when it infects neuronal cells, an important event in ZIKV pathogenesis that does not occur in Dengue. Whereas in all cell types, ZIKV virus enhances the expression of proteins, such as the cell death proteins ZBP1, RIPK 1 and 3, in neuronal cells an additional event also occurs. Thus ZIKV infected neuronal cells express immune responsive gene 1 (IRG1) whose product generates itaconate from cis-aconite, a component of the TCA cycle (31). The itaconate in turn competitively inhibits succinate dehydrogenase (SDH) thus maintaining succinate levels and this keeps neuronal cells alive (31–34). In addition, the inhibition of SDH activity inhibits ZIKV replication (31). Why the IRG1 mediated effect only occurs in neuronal cells is unclear, but could be related to the increased resistance of mature neuronal cells to apoptosis. This effect is thought to occur because neurons have some protein expression differences. These includes low level of apoptotic protease activating factor 1, increased X-linked inhibitor of apoptosis protein associated factor, and reduced caspase-3 involved in programmed cell death (35).

Hepatitis C virus (HCV) is another human pathogen that causes metabolic effects but these change at different times after infection. In the first 24 h after infection, almost all pathways used to produce the macromolecules needed for the synthesis of new viruses are elevated (36). These include both FAS and FAO with the simultaneous elevation of both FAO and FAS likely needed to provide intermediates for the TCA cycle required for virus envelop formation. In later stages (48 h), several pathways are suppressed and then the infected cell’s integrity is sustained by metabolizing host cell amino acids, especially glutamine, to sustain the TCA cycle and avoid cell death (36).

Viruses may show marked differences in the metabolic changes they impose on target cells. For example, the two herpesviruses human cytomegalovirus (HCMV) and herpes simplex virus (HSV) induce quite different effects on cellular metabolism. Infection with HCMV increases glycolytic flux, lactate excretion, FAS, and profoundly increases the level of intermediary molecules involved in the TCA cycle (37). The latter are needed so that the virus can make new viral envelopes, which with HCMV are always produced *de novo* (10). In contrast, infection with HSV, which replicates more rapidly (24 h) than HCMV (96 h), causes minimal effects on glycolysis and FAS but nucleotide synthesis is markedly affected with the process commandeered by HSV to produce new viral genomes (37). With HSV, this occurs by the virus directing the pentose pathway as well as the TCA cycle to produce the purines and pyrimidines needed to construct viral genomes (37). Compared to HCMV, HSV relies less on FAS since its envelope is mainly made from preexisting cellular envelope material.

All herpesviruses employ two lifestyles when interacting with the host. These are productive infection, which generates new virions and oftentimes results in the infected cell being destroyed. Herpesviruses also set up a state of latency, which permits the virus to remain indefinitely in the host representing the usual source of infection to others once viral reactivation and viral excretion occurs. Metabolic events during the two lifestyles are certain to be different, since latency involves minimal or even no synthesis of new viral proteins and oftentimes almost minimal effects on cellular metabolism. Productive infections, in contrast, can provoke profound changes, as already mentioned. There is also gathering evidence that changes in metabolism can be a lifestyle changing event, causing a latently infected cell to reenter the productive cycle, frequently a step needed to permit viral transmission to other hosts. This issue is under investigation in our laboratory. Some also speculate that differences in the metabolism of immune cells at local sites may influence the clinical consequences when reactivation from latency does occur (38). Such ideas have been advocated to explain why reactivation of HSV from latency is sometimes subclinical, but on other occasions results in significant tissue damage. Could it be, for example, that the deficiency of local fuels to support glycolysis would result in changes in viral pathogenesis? This concept is currently being explored. Metabolic differences between productive and latent infections have been described for other herpesviruses, some of which could account for pathogenesis changes such as contributing to neoplasia. With the herpes virus Epstein–Barr virus (EBV), some genes are expressed during latency that include LMP1, LMP2, and EBNA1. These may act to change aerobic glycolysis *via* effects on glucose uptake and metabolism. This topic is comprehensively discussed in a review by Piccaluga *et al* (39).. Inhibition of fatty acid synthesis helps to control the excessive multiplication of EBV associated nasopharyngeal carcinoma. Moreover, inhibition of hexokinase 2 (HK2) also provides a useful treatment modality for nasopharyngeal carcinoma (11, 40).

The relevance of understanding the nature of metabolic changes imposed by different virus infections is that targeted molecular therapies may be devised that can change the outcome of infection. For example, inhibiting glucose metabolism in cells infected with HIV promotes viral elimination by accelerating the death of infected cells (41). During the late stages of HIV replication FAS is elevated, which is needed for its envelope formation. Conceivably targeting lipid metabolism could also represent a means of counteracting HIV infection and this topic has been investigated (42). It was shown that inhibition of FAS with Fasnall and C75 inhibits HIV-1 replication *in-vitro*, as did Fasnall knockdown with small interfering RNA (siRNA). The outcome of such procedures *in-vivo* are not available. Currently, there is minimal information regarding manipulating metabolic pathways to influence the outcome of COVID-19 infection.

## The Effect of Interferon Induction and Metabolic Consequences on Cell Metabolism

A common outcome of many viral infection is to induce the production of one or more types of IFNs in the cells they infect. Curiously, many of the metabolic reprogramming events imposed by virus infections on cells that were discussed previously can be reversed by the IFN response. For instance, the increased energy and lipid metabolism essential for the replication of many viruses is readily reversed by IFN, with this representing one of the ways by which the IFN response acts to control virus infections (43). Characteristically, IFNs are released and bind to nearby cells that express specific receptors. This interaction signals the cell to express multiple (>400) so-called interferon stimulated genes (ISG) whose gene products express a wide range of biological activities (44, 45). One common effect is that the cell produces molecules that have antiviral activity. Other ISGs may influence cellular metabolism that in turn can affect the outcome of a virus infection. For example, a response in cells whose IFN-γ receptors are engaged is that transcription factor expression, related with steroid biosynthesis, are changed (46). Transcription levels of the sterol regulatory binding protein (SREGP), which acts to downregulate cholesterol biosynthesis, are suppressed and this limits the availability of lipids which are needed particularly to generate new enveloped viruses. The SREGP can also inhibit cell entry of some viruses such as HIV-1 and it also impairs intracellular virus budding through the endoplasmic and cell membranes (47, 48).

**Figure 1:**
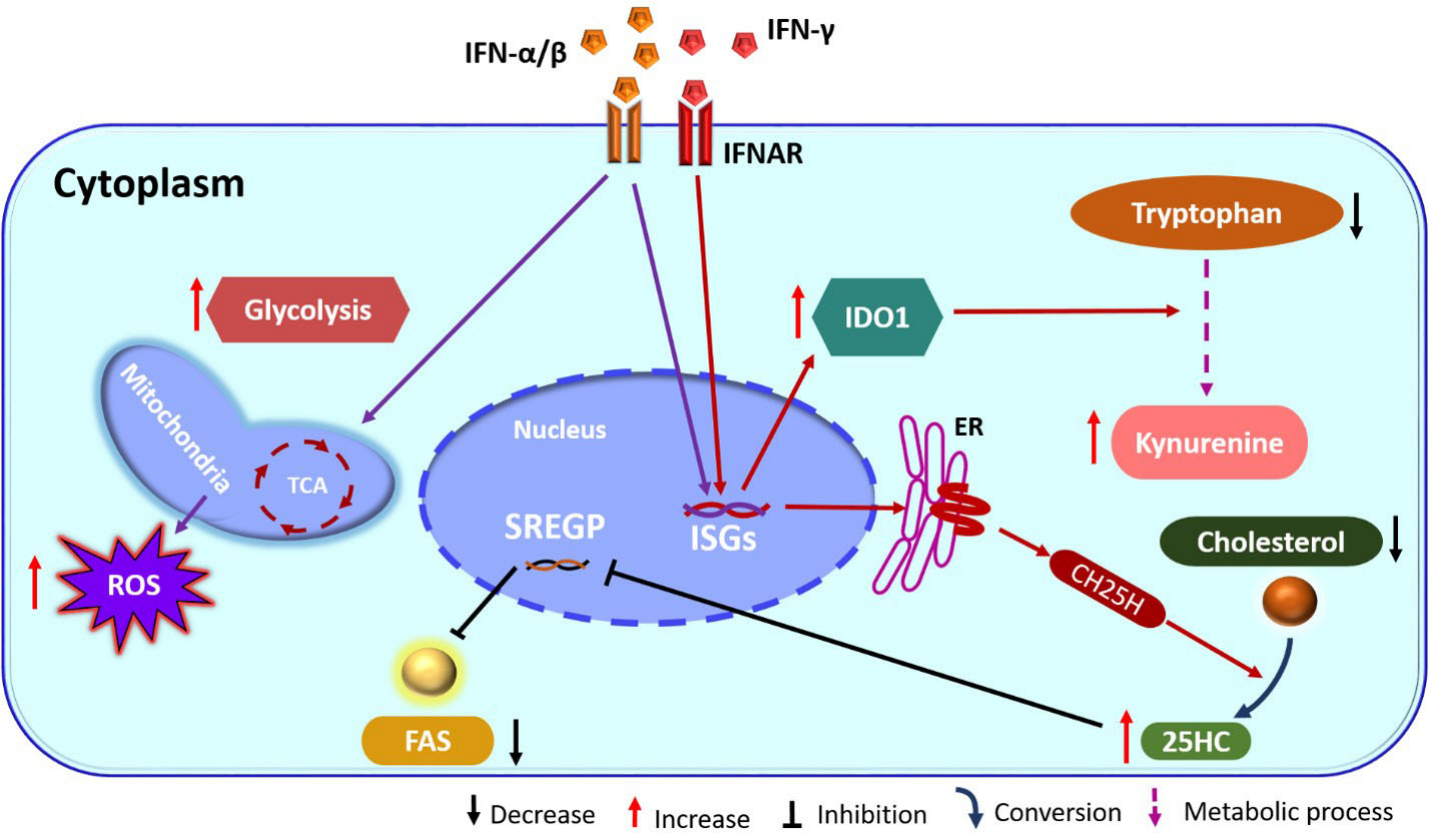
IFN action on cellular metabolism pathways. IFN binds its receptor (IFNAR) and induces ISGs, which includes the enzyme CH25H. This enzyme catalyzes the formation of 25HC from cholesterol. 25HC has inhibitory action on SREGP and as a consequence FAS is reduced. In addition, IFN also stimulates TCA and glycolysis. Stimulation of ISGs also increase IDO1 and enhances the kynurenine pathway from tryptophan (ER, endoplasmic reticulum; IFN, interferon; IFNAR, IFN-receptor; TCA, tricarboxylic acid cycle; CH25H, cholesterol 25-hydroxylase; ISGs, interferon stimulated genes; 25HC, 25-hydroxycholesterol; SREGP, sterol regulatory binding protein; IDO1, indoleamine 2,3 dioxygenase1; FAS, fatty acid synthesis; ROS, reactive oxygen species).

Another ISG induced by type I IFN, that affects cellular metabolism encodes the enzyme cholesterol-25-hydroxylase (CH25H). This enzyme converts cholesterol to soluble oxysterol 25-hydroxycholesterol (25HC) that in turn serves to decrease cholesterol accumulation within cells (**Figure 1**). The overall effect is increased resistance to several viruses such as ZIKV and other flaviviruses as well as with some togaviruses (49, 50). The inhibitory effect is the consequence of inhibition of viral fusion needed to enter and transport within cells as well as for the formation of new virions (51). Upon administration to animals, the molecule 25HC can also exert antiviral effects. For example, the pups of pregnant mice given 25HC are protected from the neurological consequences of ZIKV infection (49). In addition, in humanized mice treatment with 25HC increased resistance of their T cells to HIV infection (51). Finally, by *in-vitro* studies 25HC was shown to have antiviral effects against several viruses that include vesicular stomatitis virus (VSV) and HSV from *Herpesviridae*, HIV from *Retroviridae*, Ebola virus (EBOV) from *Filoviridae*, Rift Valley fever virus (RVFV) from *Bunyaviridae*, Russian spring-summer encephalitis virus (RSSEV) *Flavirviridae*, and Nipah virus within *Paramyxoviridae* family (51).

Other ISGs can also influence lipid metabolism which then acts to limit infection and/or change the inflammatory function of cells such as macrophages. For example, monocytes exposed to IFN-α change their lipid metabolism and undergo enhanced reactive oxygen species (ROS) production, which is essential for the antiviral effector functions of macrophages (52). Another critical influence on the outcome of virus infection set off by IFN-γ triggering is induction of the enzyme indoleamine 2,3 dioxygenase (IDO) (**Figure 1**) (53). This effect is usually mediated by IFN-γ and occurs mainly in macrophages and dendritic cells. The IDO enzyme converts tryptophan to kynurenine, which has several effects on immune function. These include inhibitory effects on some innate immune activities and suppression of T cell proliferation and function (54). Cells with upregulated IDO also increase their uptake of tryptophan that acts also to inhibit the effector activity of T cells (54). IDO may also have direct inhibitory effects on some viruses that include HSV (55), HCMV (56) and human parainfluenza virus type 3 (57) perhaps mediated by depriving infected cells of adequate tryptophan. Some have suggested tryptophan deprivation could also be one means by which herpes simplex virus type 2 latency is maintained and the lytic cycle suppressed (58).

## Metabolic Diseases and the Outcome of Virus Infections

As discussed in the previous section, when most viruses infect target cells they induce changes in metabolism that are needed for the virus to be replicated. Examples includes HCMV which upregulate glycolysis in cultured cells (10) and KSHV, which upregulate glutaminolysis in microvascular endothelial cells (18). A complete list of metabolic changes caused by various viruses is given in **Table 1**. Accordingly, one expects that, in situations where an aspect of host metabolism is malfunctioning, because of genetic or extrinsic reasons, the outcome of a virus infection may be affected. This topic has been mainly explored with diabetes mellitus (DM), which affects sugar metabolism. There are several reports that show that immune defense mechanisms may be less effective as a consequence of diabetes so increased susceptibility to infections is to be anticipated (59–61). Several innate immune activities are diminished during diabetes. These include reduced levels of the antibacterial molecule beta defensin (62), reduction in neutrophil traps (63), reduced capacity to undergo the respiratory burst (64), and to generate antimicrobial reactive radicals such as superoxide (65). In DM, reduced production of some enzymes such as elastase and myeloperoxidase also occur and monocyte phagocytosis may be compromised as well (66, 67). All suppressive effects observed on innate defenses during DM may be caused by hyperglycemia, but in most instances, details of molecular events involved still need to be elucidated. In the case of beta defensin production, reduced levels during diabetic hyperglycemia were attributed to the production of dicarbonyl methylglyoxal from glucose, which changes the surface charge of beta defensin and reduces its antibacterial activity (62). The effects of DM on innate immunity explains why many bacterial infections are more common during DM, but also increased susceptibility to some virus infections has been observed (68). Most reports about changed susceptibility to virus infection in DM patients involves influenza (Flu) but it has recently become evident that diabetics (both the mostly studied type II as well as type I) may experience more severe consequences of COVID-19 infection (69, 70). Rising evidence also shows that COVID-19 infection may worsen the signs of diabetes (71) and also the infection may cause the onset of DM (72).

During flu epidemics, diabetics may experience more clinical problems than non-diabetics as noted in several studies (73, 74). Thus, diabetics require more frequent hospitalization and adult diabetics suffer more severe consequences and higher mortality rates than non-diabetics (75, 76). However, it is far from clear as to the mechanisms which explain increased lesions, although decreased activity of one or more immune components is usually advocated (77–80). Another confounding issue is defining which aspect of DM accounts for susceptibility. Several studies link suppressed immunity with hyperglycemia and others to immune problems caused by obesity, which is a common outcome particularly with insulin resistant type 2 DM. The immunological consequences of obesity as regards effects on the pattern of viral infections is further discussed in a later section.

A better understanding of how DM can cause increased susceptibility to Flu comes from studies of type 1 DM in mouse models, which do show enhanced susceptibility (81). Mice with DM may develop higher levels of virus in their lungs compared to normal mice (82). In one model, the increased susceptibility was associated with excessive levels of glucose which acted to block the function of a surface protein lectin (SP-L) on endothelial cells that normally plays a protective role against Flu. SP-L may also act on neutrophils changing many of their functions and their ability to bind to virus.

In a drug induced model of type 1 DM, diabetic mice compared to non-diabetic controls had higher numbers of infected lung epithelial cells and developed more severe lung and pancreatic damage upon infection with several subtypes of influenza A virus (H1N1, H5N1 and H7N2) (83). In addition, higher mortality levels occurred upon infection with virulent strains of H1N1 as well as with the H5N1 subtype (83). However, this study provided no mechanistic explanation for the findings.

As we write this review, we are deep into the COVID-19 pandemic which raises the issue if diabetics are prone to develop more severe consequences of infection. Several reports indicate this is happening. For instance, in a study in China, out of 52 persons admitted to the intensive care unit (ICU), 32 died and 22% of these patients were diabetics (69). In another study of 173 patients, 16.2% were diabetics and these experienced severe disease consequences (84). In a study of 1,527 patients, two-fold higher numbers of diabetics required admittance to the ICU than normal patients (85). In New York, a recent study reported that, out of 5,700 COVID-19 patients, 33.8% had a history of diabetes which compares to around 10% in the uninfected population with diabetes (86). Thus, evidence accumulates to show that susceptibility to COVID-19 infection is increased in those with diabetes, but so far a mechanistic understanding of why this occurs remains to be shown. However, conceivably one explanation might be associated with the main receptor COVID-19 uses to enter cells, the angiotensin-converting enzyme 2 (ACE2). Curiously, patients with diabetes type 2 upregulate ACE2 at least in renal tissues (87, 88) and expression is further increased upon treatment with ACE inhibitors (89, 90). It is not clear in humans if ACE2 is upregulated in lung tissue where COVID-19 replicates, but in diabetic mice ACE2 levels are increased in lung tissues compared to healthy controls (91, 92). The protein component of COVID-19 that binds ACE2 is the spike protein and soluble forms of ACE2 can block virus infection (93, 94). Finally, trials are underway to determine if ACE2 inhibitor drugs such as the commonly used drug to control high blood pressure, losartan, has any beneficial effect on the outcome of COVID-19 infection in healthy patients. Very recently, it was observed that the incidence of Flu, which uses the ACE2 receptor to mediate lung damage, was reduced in persons that used ACE2 inhibitor drugs to control their blood pressure (95–97). Moreover, prolonged drug use improved the outcome of the Flu infection (95). For the expanding knowledge concerning COVID-19 and DM and management of this interaction, an excellent review was recently published (72).

Other coronaviruses associated with human disease may also be more severe in diabetics. For example, in a study in China of 135 persons that died of severe acute respiratory syndrome (SARS), 21.5% were diabetic, whereas in 385 survivors only 3.9% had diabetes (98). With Middle East respiratory syndrome (MERS) patients in the Middle East, 50.9% of those who died were diabetic, whereas in those that recovered only 22.9% were diabetics (99). In the case of MERS, experimental studies were done in diabetic and non-diabetic control mice. The diabetic animals had more extended disease, diminished innate immune responses, and also had lower T cell responses to infection (100). It is also worth mentioning that with the SARS coronavirus, which has many similarities to COVID-19, there is evidence that the infection could damage islet cells and help precipitate DM (101). Additionally, very recently COVID-19 was advocated to upregulate ACE2 receptors on pancreatic cells making them a target for COVID-19 infection (102).

In addition to coronaviruses and flu, other virus infections may have a different, usually more severe outcome in diabetic compared to non-diabetics. One example is HSV-1 infection which causes increased facial nerve damage in diabetic mice after auricular infection (103, 104). There is also the suggestion that being diabetic is a risk factor for developing herpes zoster caused by varicella-zoster virus (VZV) (105), perhaps explained by diminished T cell responses in DM patients.

It would be also interesting to know if the outcome of chronic virus infections such as HIV and hepatitis are more consequential in those with diabetes (106). There is surprisingly little information on that topic, but what has been more investigated is whether HIV infection or treatments used to control HIV can act as triggering factors to set off DM. For example, one study on HIV-seropositive patients treated with highly active antiretroviral therapy showed that four times more patients developed type 2 DM than occurred in the HIV-seronegative group (107). Treatment of HIV with protease inhibitor drugs can also result in developing DM, perhaps explained by the inhibitory effects of the drugs on proteins involved in glucose metabolism such as Glut-4 (108) and cellular retinoic acid–binding protein type-I (109). Additionally, protease inhibitors may hamper pancreatic beta cell function (110) and also reduce insulin secretion (111). However, it seems that the issue of whether diabetics are more prone to develop chronic viral infections requires more research.

Obesity can also be considered as a metabolic disease and evidence accumulates to show that the outcome of a virus infection can differ in obese compared to non-obese humans and animals (112, 113). The topic of obesity and its influence on the outcome of virus infection has been reviewed (114). Reports document that severely obese persons may respond poorly to several vaccines and generate reduced antibody and T cell responses to infection and vaccination (115). For example, in the USA during the 2009 H1N1 pandemic, morbidly obesity patients (i.e., body mass index ≥ 40) were more prone to hospitalization and death (116) than non-obese persons. In China in the same pandemic, 19% of 9,966 patients admitted to the ICU were diabetic and many died (117). We are also realizing that obesity may predispose persons to more severe consequences of COVID-19 infection. For example, in China obese persons had a 2.42 fold higher chance of developing pneumonia upon COVID-19 infection than did the non-obese (118). Similarly, in France obese persons infected with COVID-19 had higher rates of admission to the ICU and greater needs for mechanical ventilation, outcomes directly proportional with their increased body mass indices (85). In a New York study involving 5,700 COVID-19 patients, 41.7% were obese (86). Explanations why obese persons become more susceptible to COVID-19 infection needs to be established. However, one consequence of obesity is increased expression of the ACE2 receptor on adipocytes making these cells potent targets for COVID-19, which could serve to increase the viral load (119, 120). Additionally, there are several changes in immune function that occur in obese subjects. These include higher ratios of M1:M2 macrophages (121), reduced T cells but elevated B cell numbers in lymph nodes (122) decreases bone marrow hematopoiesis (123) and reduced production of IFN-γ, TNF-α, IL-6, TGF-β1 by T cells (124).

Studies in mice models of obesity have revealed mechanisms that could explain their increased susceptibility to virus infection. These include effects on N-acyltransferase metabolism, fatty acid pathways (10-fold increase in 3-oxododecanoic acid and 4-hydroxyisovaleric acid) and nucleotide metabolism pathways. In obese mice, Flu can induce more oxidative stress than in lean animals and mice develop diminished T cell memory responses to infection (125).

One could anticipate that diseases of the thyroid gland, either hypothyroidism or Graves’ disease (hyperthyroidism), might be associated with changed responses to infections since thyroid hormones (TH) have a significant effect on general metabolism. For example, TH can affect liver enzymes involved in FAO and also impact on the uptake of free fatty acids by muscle and fat cells (126). Additionally, some have shown that TH can directly influence glucose utilization (127, 128). No reports have directly linked TH dysfunctions with changed susceptibility to a virus infection. However, some observations have tried to associate thyroid disease problems with the control of latency with some herpesviruses. One topic for evaluation has been herpes zoster, the lesion that occurs at a neurodermatome when VZV reactivates from its latent infection state (129). One study reported that when TH levels were low herpes zoster lesions were observed more frequently and showed that lesion outbreaks were less frequent in TH treated patients than in untreated persons, although differences did not reach statistical significance (130). One might also anticipate, based on mouse studies which showed that the TH receptor can influence the expression *in-vitro* of the latency associated transcript involved in HSV latency, that breakdown of HSV latency could be affected by TH levels, but such an effect has not been reported (131).

## Influence of Nutrition on Outcome of Virus Infections

In today’s world, many persons adjust their diet to lose or gain weight. They may also favor one form of diet over another, or use non-prescription herbal remedies. The objective is usually to improve their health and well-being, but evidence for the success of such maneuvers is less than compelling, at least when it come to the outcome of human virus infections. However, it is generally agreed that malnutrition, especially marked calorie deficiency, can increase the susceptibility of children to virus diseases such as measles and respiratory syncytial virus (132, 133). There is also evidence that vitamin A deficiency, which is prevalent in malnourished children in South-East Asia, results commonly in increased susceptibility to measles as well as to both respiratory and enteric viral infections. One explanation could be that several cells of the immune system particularly dendritic cells and T cells are activated and hence more protective when their promoter regions bind to the vitamin A derivative retinoic acid (134–136). Other ideas include an effect of the vitamin A metabolites changing the nature of inflammatory reactions to became less tissue damaging *via* effects on regulatory T cells (137). In several studies, supplementation with vitamin A may increase the recovery rate and reduce mortality from measles (138, 139). Treated children develop both improved T cell and antibody responses (140). Vitamin A supplements are effective, but too expensive for general use in resource poor countries. However, a solution could be the use of genetically modified cereals such as golden rice that produce vitamin A precursors. This approach is resisted in most societies for non-scientific reasons.

Our most complete understanding of how nutrition can influence the outcome of virus infections comes from diet manipulation studies in animals, most commonly Flu infections in mice. Several different approaches have been used. These include comparing the outcome of a virus infection in animals or the offspring of pregnant mothers that received a so-called Mediterranean diet (~10% fat) *vs*. a Western (~40% fat) diet, comparing the effect of feeding diets with different fat levels and fat composition, as well as differences in fiber content (141). The consequences of nutritional changes on viral pathogenesis are often mediated by the effects of dietary intake on the composition and activity of the enteric microflora, a topic of intense interest at present. Most studies conclude that a high fat diet (HFD) can result in more severe Flu and higher mortality than occurs in recipients of a low fat diet (LFD), or normal chow diet (NCD). For example, in a study comparing responses to the pandemic Flu strain H1N1 in mice that received either a HFD, LFD, or NCD, significant differences were observed (142). Mice fed the HFD suffered 80% mortality along with enhanced lung destruction, inflammation and excess inflammatory cytokine and chemokine responses. Recipients of the LFD and NCD had mortality rates of 40 and 0% respectively. Compared to the other groups, the HFD mice showed marked changes in some metabolites in their blood. These included metabolites related to N-acyltransferase metabolism, fatty acid pathways (10-fold increase in 3-oxododecanoic acid and 4-hydroxyisovaleric acid) and nucleotide metabolism pathways. The recipients of the HFD also showed evidence of high oxidative stress, such as elevated oxidized glutathione in the lungs and the presence of high taurine levels in the urine (142). Even LFD recipients were more susceptible to influenza infection compared with mice on the NCD diet, perhaps explained by low fiber levels in the LFD (142).

The level of fat is not the only factor that affects Flu susceptibility; the chemical nature of fat in the diet also has an influence. Studies have shown that fatty acid chain length and major composition of either omega 3 or omega 6 FA (fatty acid) can also have notable effects. With regard to the latter, a diet high in omega 3 FA favors the production of anti-inflammatory activities, such as proresolving mediator synthesis (143), whereas diets rich in omega 6 FA favors proinflammatory mediator production such as prostaglandins and leukotrienes (144). Omega 3 predominance is beneficial to control inflammatory diseases that include situations where virus lesions result from a reaction dominated by inflammatory cytokine producing T cells (143). However, with viruses that cause disease by direct effects, the omega 3 rich diet may increase susceptibility. This has been noted with Flu where an omega 3 rich diet, such as feeding fish oil, results in increased severity and mortality following intranasal infection (145). The recipients developed reduced NK cell and neutrophil responses as well as reduced inflammatory mediator production involved in viral control (145). Their CD8 T cell responses were also reduced compared to animals fed the control diet.

Many studies have shown that the chain length of FAs included in the diet can play a major role in affecting the expression of inflammatory lesions caused by viruses (146, 147). Most studies have focused on the influence of fiber composition since high fiber diets generate an abundance of short chain fatty acids (SCFA) as a consequence of microbial digestion in the gut. However, feeding a SCFA such as butyrate as a dietary supplement can also boost the resistance of mice to Flu challenge, particularly in the early stages of infection. In one study, whereas all control mice were dead by 8 days’ post infection (PI), those fed additional butyrate showed 100, 75, and 40% survival rates at 8, 12, and 18 days’ PI respectively (147). The main effect of SCFA feeding is to expand anti-inflammatory aspects of the immune response that include Treg and M2 macrophages.

As mentioned previously, the outcome of a virus infection can be changed if animals are given a high fiber diet. This has been observed with Flu, HSV, and RSV infection all of which cause less disease when animals receive a high fiber diet (146–148). The effect is now generally accepted to be explained by the expansion of bacteria such as *Bifidobacteria* and *Bacteroides* species in the gut microflora that metabolize fiber into SCFA (butyrate, acetate and propionate) (147). The resulting protective effect against flu was the consequence of an increase in the number and activation status of CD8 T cell responses and effects on the bone marrow that favored M2 macrophage differentiation, cells which produce less tissue damaging cytokines and chemokines (147). The SCFA molecules mediate such effects by binding to specific fatty acid receptors on responding cells. One consequence is an increase in mitochondrial mass and an increase in metabolites for the TCA cycle resulting enhancement of the OXPHOS energy pathway (147).

In the case of respiratory syncytial virus (RSV), protective effects were observed in mice that received a high fiber diet as well as a diet supplemented with acetate (146). In those experiments, the protective effects were attributed to the enhanced production of IFN-β from lung epithelial cells, serving to inhibit viral replication. High fiber diets may also protect against the inflammatory effects of HSV infection (148). In this instance, the protective effects were attributed to the generation of a more effective CD8 T cell response which included an expansion of memory cells to counteract future infection. Recently, our laboratory has shown beneficial effects of supplementing the diet with sodium propionate (SP) to reduce the tissue damage caused by HSV-1 infection in the eye (149).

Other studies on the effects of diet on the outcome of virus infections have evaluated the influence of supplementing the diet for certain amino acids. For example, the effect of additional glutamine and leucine has been shown to influence the outcome of vaginal infection of mice with HSV (150). Mice receiving the additional amino acids had reduced viral burdens and less mortality than those on the control diet. The supplement recipients also had elevated NK cell and IFN-γ producing CD4 Th1 T cell responses (150). Adding glutamine to the diet could also influence the stability of HSV latency. This might occur as a consequence of HSV specific CD8 T cells being expanded in the latently infected trigeminal ganglia where they are thought to function by prevented reactivation from latency (151). Another interesting observation on the value of SCFA feeding was that this could reduce the consequence of secondary bacterial infections which commonly cause Flu-associated respiratory infections to be more severe. Some have reported that a consequence of flu infection can be a change in bacterial types present in the gut, although it is not clear mechanistically how this dysbiosis occurs. The outcome is reduced presence of those bacteria species that generate SCFA especially acetate, an effect overcome by SCFA feeding (152).

Finally, with regard to nutritional influences on viral infections is the interesting observation that feeding additional glucose or SCFA can overcome the detrimental effects that high ambient temperature may exert on susceptibility to some virus infections, an issue expected to become more problematic in a warming world. Thus, when influenza infected mice were kept at 36°C compared to room temperature, they showed more susceptibility and mounted impaired immune responses that included reduced virus specific CD8 and CD4 T cells, IgG levels, and reduction in several inflammatory cytokines. The outcome of high temperature exposure was overcome either by feeding extra glucose or supplementing the diet with additional SCFA (153). Impaired immunity at high temperature has also been observed with ZIKV and Bunyavirus infections (153).

## Manipulating Metabolism to Reshape the Outcome of a Virus Infection

**Figure 2:**
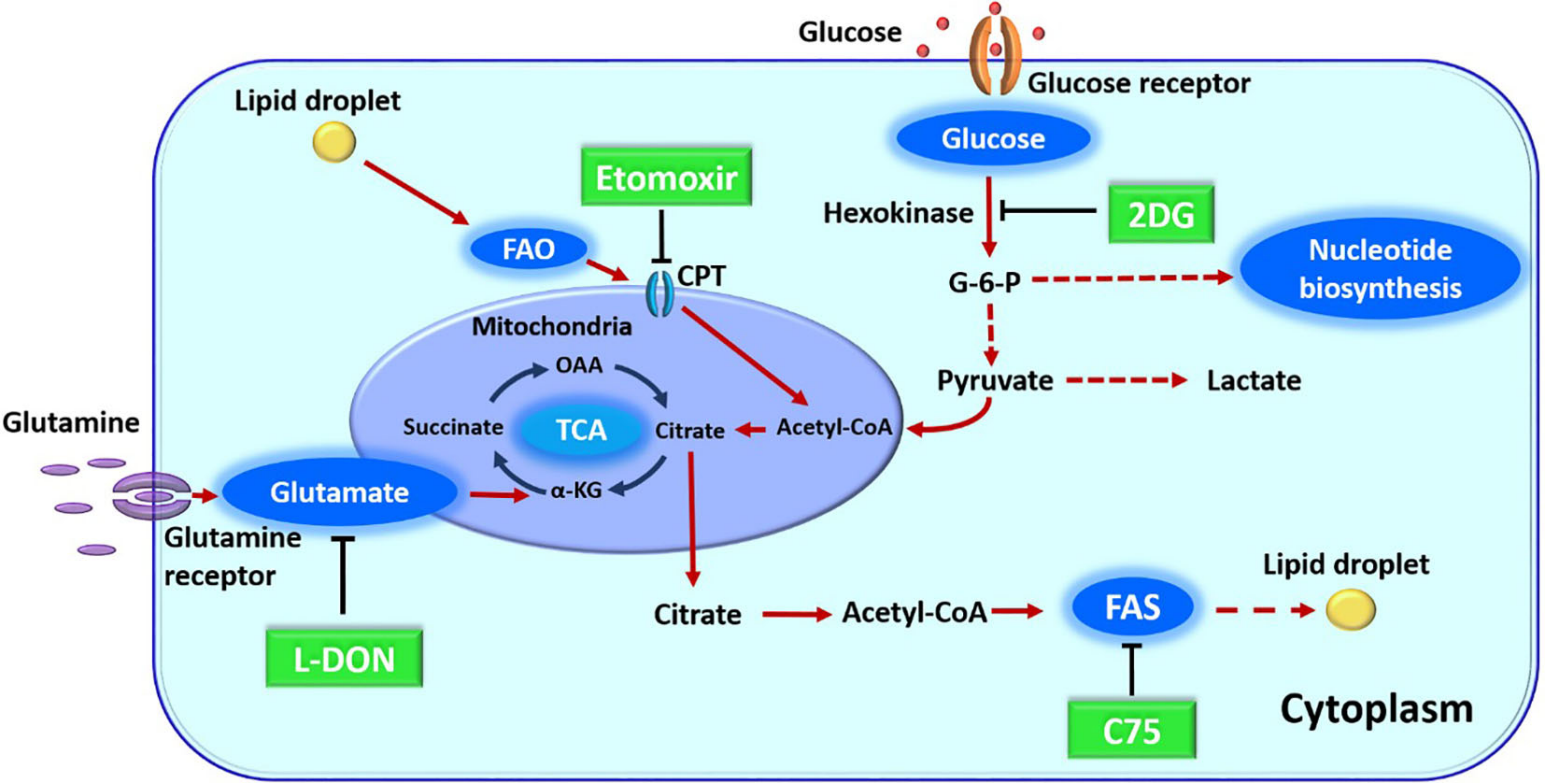
Major metabolic pathway along with their inhibitors. 2DG (2-deoxy-D-glucose) blocks glycolysis. L-DON (6-diazo-5-oxo-L-norleucine) blocks glutaminolysis by acting on glutamate. Etomoxir blocks FAO (fatty acid oxidation) by its inhibitor action on CPT1a (carnitine palmitoyltransferase), C75 (fatty acid synthase inhibitor) blocks fatty acid synthesis (FAS) by inhibiting a fatty acid synthase enzyme. OAA, oxaloacetic acid; alpha KG, alpha-ketoglutarate; G-6-P, glucose 6-phosphate; TCA, tricarboxylic acid cycle.

In previous sections, we have discussed how metabolic pathways can be changed upon virus infection and how host intrinsic metabolic activities and extrinsic events that affect metabolism can influence the expression of infection. These situations raise the issue as to whether manipulating one or more aspects of host metabolism represent useful and perhaps convenient approaches to control the outcome of virus infections. They might be especially useful with infections not well controlled by vaccines or antiviral drugs. Currently, the majority of reports that target metabolism for disease control have dealt with autoimmunity or cancer, but viral diseases especially chronic persistent infections, could be a fruitful field for investigation. With respect to virus infections, the great majority of studies on metabolic manipulations have been performed *in-vitro* with various drugs (**Figure 2**) and many of these are recorded in **Table 2**. As mentioned *in-vivo* studies are sparse. An early report came from the Medzhitov laboratory which was designed to compare the effects of calorie restriction on some viral and bacterial infections. The study showed that anorexia made neurogenic flu infections more severe, but protected against bacterial sepsis infections. Moreover, nutritional supplementation with glucose was detrimental to bacterial disease, but protected against lethal flu infection. In their models, the nutritional effects were explained not by effects on immune function, but by an ER stress effects on the brain with this causing more apoptosis in neuronal cells (167).

**Table 2:** Effect of the metabolic inhibitor drug on various virus infected cell line.

Virus	Nucleic acid	Metabolism targeted	Drug used	Cell line	Outcome	Reference
KSHV	DNA	Fatty acid synthesis	TOFA	Tert-immortalized microvascular endothelial (TIME) cells	Decrease the production of virion	(154)
Marek’s disease virus			C75 and TOFA	Chicken embryonic fibroblast 396 cells (CEFs)	Reduce virus replication	(155)
Vaccinia virus			TOFA and C75	BSC40 cells	Reduction in virus	(156)
HCMV			TOFA and C75	Fibroblasts/MDCK cells	Reduce viral replication	(10)
Chikungunya virus	RNA		C75 and cerulenin	HeLa cells	Decrease the replication of virus	(157)
HIV			Fasnall and C75	TZM-bl cells	Inhibit viral replication	(42)
WNV			C75 and cerulenin	Huh-7 cells and vero cell	Decrease the production of virus	(158)
Usutu virus			TOFA	Huh-7, vero and Neuro2a cell		(159)
DENV			C75 and cerulenin	Huh-7.5 cells	Reduction in DENV replication in dose dependent manner	(22)
Influenza A			TOFA and C75	Fibroblasts/MDCK cells	Reduce viral replication	(10)
Hepatitis C virus			C75	Huh7 and Huh7.5.1 cells		(21)
DENV	RNA	Fatty acid oxidation	Etomoxir	Huh-7 cells	Decrease in virus in dose dependent manner	(160)
Vaccinia virus	DNA			BSC40 cells	Reduction in virus	(156)
H1NI influenza A virus	RNA	Glycolysis inhibition	2DG and 3-BrPa	MDCK cells	Decrease viral replication in dose dependent manner	(161)
DENV			2DG and Oxamate	human foreskin fibroblasts	Reduce DENV replication	(15)
Human retrovirus HTLV-1			2DG	PBMCs	Inhibition of transcription of HTLV-1	(162)
Rhinoviruses				Hela cell, fibroblast	Inhibit viral replication	(163)
KSHV	DNA		Oxamate	TIME and iSLK cells	Reduce viral production	(154)
HSV-1	DNA	Glutamine inhibition	L-DON	Vero cells	Anti-viral effect	(164)
VSV	RNA			Vero, LLCMK2, BHK and primary monkey kidney cells		(165)
Human parainfluenza virus type 2						
Mumps viruses						
Human respiratory syncytial virus				CV-1 cells		(166)

TOFA, 5-(tetradecyloxy)-2-furoic acid; 3-BrPa, 3-bromopyruvate; MDCK cells, Madin-Darby canine kidney cells; PMBCs, peripheral blood mononuclear cell; 2DG, 2-deoxy-D-glucose; L-DON, 6-diazo-5-oxo-L-norleucine; LLCMK2, rhesus monkey kidney epithelial cells; BHK, baby hamster kidney fibroblasts; CV-1 cells, normal African green monkey kidney fibroblast cells.

Another study looked at the *in-vivo* consequences of interfering with glucose utilization using 2DG (2-deoxy-D-glucose) therapy in a viral disease model (168). Inhibition of glucose utilization resulted in diminished HSV induced inflammatory lesions in the eye (168). The protective therapy was explained by a reduction in proinflammatory T cell activity that primarily use glycolysis to supply their energy. In this study, the Treg responses, which were shown previously to suppress the severity of ocular lesions (168), was not affected by 2DG therapy. Accordingly, metabolic manipulation, such as reprogramming glucose metabolism, can represent an approach to control the expression of viral inflammatory disease. Other reports on this topic have also appeared (163).

Another potential metabolic target that has promise to control the extent of a virus infection is fatty acid metabolism which is a crucial event for many viruses such as flaviviruses that depend on cellular lipids to complete their life cycle. Success at controlling several human flavivirus disease agents has been achieved *in-vitro* using inhibitors of acetyl coenzyme carboxylase, which is essential for *de novo* lipogenesis (169). So far studies on *in-vivo* effects have only been done using a mouse model of West Nile fever (WNF). In such studies, the infection was successfully controlled including lesions in the kidney (169). The latter is relevant since renal complications are a prominent feature of persistent WNF infection in humans (170).

Another metabolic step accessible for inhibition to suppress viral diseases is glutamine metabolism. Thus this amino acid is used to support proliferation and cytokine producing activity of inflammatory T cells and in addition is a precursor of a neurotoxic molecule, glutamate. Inhibiting glutamine metabolism was shown to be valuable to diminish CNS inflammatory lesions caused by Sindbis virus infection (171). Inhibiting glutamine metabolism could well be an approach to control other immunoinflammatory viral lesions and our group is exploring its values to limit herpetic stromal keratitis lesions.

We anticipate that additional approaches to manipulate metabolism will be useful to shape the outcome of virus infections particularly when used along with other therapies such as antiviral and anti-inflammatory drugs.

## Conclusions

Controlling virus infections is most effectively achieved using vaccines and with a few viruses specific anti-viral drugs. However, such approaches are not available for many infections, which includes COVID-19, the cause of a current pandemic. We need new approaches to control virus infections and this review focusses on changing the metabolic events that occur during viral infections. We advocate that manipulating metabolic activities represent a useful approach to control the outcome of some viral infections that may include COVID-19 infection. We described how metabolic changes are set into play by different viral infections and point out that changing the metabolic environment might be one means of controlling if the virus host relationship is productive and tissue damaging or inapparent. We also review how the outcome of virus infections can be affected by the metabolic status of the host. Particularly relevant are DM and obesity that impact on the clinical consequences of infections such as Flu and COVID-19 infection. In addition, the nutritional status of the host may also influence the expression of some virus infections and changing the diet holds promise as one way to control the outcome of some viral infections. The major question addressed was whether it is possible to reshape the outcome of a viral infection, particularly those where the host response contributes to tissue damage. The topic has received minimal investigation, but some studies do show that controlling events such as glycolysis, glutaminolysis and fatty acid metabolism are showing promise as an approaches to limit the severity of some viral infections. We presume that manipulating metabolism to reshape the nature of some virus infections will become a valuable addition to the current approaches available to control virus infections.

## Author Contributions

BR conceptualized the manuscript. BR, DS, EB, and MM wrote the manuscript. All authors contributed to the article and approved the submitted version.

## Funding

This work was supported by the NIH 2020 R21 AI (number: 5R21AI142862-02) and NIH 2020 R01 (number: EY5R01EY005093-35).

## Conflict of Interest

The authors declare that the research was conducted in the absence of any commercial or financial relationships that could be construed as a potential conflict of interest.
